# Detection of Fatigue Cracks for Concrete Structures by Using Carbon Ink-Based Conductive Skin and Electrical Resistance Tomography

**DOI:** 10.3390/s23208382

**Published:** 2023-10-11

**Authors:** Chenning Cai, Shaolin Chen, Lina Liu

**Affiliations:** College of Civil Aviation, Nanjing University of Aeronautics and Astronautics, Nanjing 211106, China; caichenning@nuaa.edu.cn (C.C.);

**Keywords:** crack detection, concrete structure, electrical resistance tomography, conductive sensing skin, carbon ink

## Abstract

Concrete is among the most widely used structural materials in buildings and bridges all over the world. During their service life, concrete structures may inevitably display cracks due to long-term fatigue loads, leading to the degradation of structural integrity. Thus, it is very important to detect cracks and their growth in concrete structures using an automated structural health monitoring system. In this paper, experimental research on crack detection and imaging of concrete structures by using sensing skin and electrical resistance tomography (ERT) is presented. Carbon ink is screen-printed on the surface of concrete as a conductive material to form sensing skins. With these sensing skins, when cracks occur on or near the surface, it breaks the continuity of the sensing skins and significantly reduces conductivity in cracking areas. Then, after exciting small currents in sensing skins and measuring related voltage data, an inverse analysis based on total variation (TV) regularization is adopted to reconstruct tomographic images showing conductivity changes in sensing skins, to detect the occurrence and growth of cracks. The effectiveness of conductive sensing skins and our related crack detection method is validated in experimental studies on a concrete beam subjected to fatigue tests.

## 1. Introduction

Concrete is among the most widely used structural materials in infrastructures, such as buildings and bridges, all over the world. During their service life, concrete structures may inevitably display different types of damage, leading to the degradation of structural integrity. Among the different types of damage, cracking is a major factor in the premature deterioration of concrete structures, which seriously affects the service life of structures. Cracks provide erosion paths for water and corrosive agents to enter the interior of the concrete structures, accelerating the degradation of concrete structure performance [1,2,3]. Therefore, the timely detection of cracks and obtaining information on their location and length are crucial for evaluating the safety of concrete structures and predicting their service life [4,5].

During the past several decades, non-destructive inspection/evaluation (NDI/E) techniques, like ultrasound [6], acoustic emission [7], and X-ray tomography [8], have been developed to detect cracks in concrete. However, these traditional NDI/E techniques usually depend on bulk and expensive instruments, they are difficult to perform in outdoor environments, and they are not suitable for long-term online monitoring [9]. Compared with these methods, methods based on electrical measurements are generally attractive because they can be performed quickly and costs are relatively low [10,11,12,13]. Among electrical methods, electrical resistance tomography (ERT) technology can provide visualization results of damage that are convenient for obtaining quantitative information, such as the damage location and damage size. These methods have gradually attracted the attention of researchers in civil engineering [14,15,16,17]. For instance, Karhunen et al. [18] applied ERT technology to conduct 3D imaging of crack defects in concrete slabs and beams, and achieved three-dimensional visualization of the damage. Their results demonstrated that it is completely feasible to use ERT to detect and identify damage in concrete; the technology cannot only locate damage locations but also characterize crack plane morphology and depth. Ren et al. [19] conducted research on ERT technology for various defects in concrete by using embedded electrodes and considering the influence of hydration reactions. They showed that ERT has broad application prospects in concrete defect detection and on-site monitoring, but concrete materials are poor conductors with low and uneven conductivity, leading to poor tomography results when ERT technology was directly applied to ordinary concrete. In recent years, with the development of material science, different conductive materials have been added to concrete structures to enable self-sensing. For example, Nayak and Das incorporated metallic waste iron powder into concrete mortars and used ERT to reconstruct conductivity images for the configuration of damage [20]. Gupta et al. sprayed multi-walled carbon nanotubes (MWCNTs) onto dried aggregates and fabricated concrete pavements with self-sensing functions, then ERT was employed to characterize spatially distributed damage when conducting accelerated tests [21].

In addition to the aforementioned studies, considering the fact that the observation of concrete cracks is mainly conducted on the surface, researchers have proposed different types of conductive sensing skins, which can be integrated into the concrete surface to detect surface damage by monitoring changes in conductivity or resistivity. For example, Loh et al. [22] carried out work on attaching a carbon nanotube (CNT)-based film to the surface of cement-based materials, and then monitored the conductivity of the CNT film by using ERT when applying loads to the substrate and producing cracks. Hallaji et al. [23,24] sprayed a thin layer of conductive silver paste material on to the surface of concrete as a sensing skin to detect and locate cracks and damage on the concrete substrate. Seppanen et al. [25] further developed a multifunctional sensing skin where conductive copper and silver were painted on the two sides of an insulation thin film to detect and localize the ingress of chlorides and cracking, respectively.

In this paper, a kind of carbon ink is proposed to screen print onto concrete surfaces as smart sensing skins to detect and identify cracks with ERT. Upon excitation of small currents in smart sensing skins and the generation of related voltage data, a total variation (TV) regularization-based algorithm is employed to generate tomographic images of conductivity changes in sensing skins caused by cracking, so as to determine the existence and development of concrete cracks in the whole process. Experimental studies on a concrete beam subjected to a fatigue test were performed to validate the effectiveness of the conductive sensing skin and the related crack detection method.

## 2. Electrical Resistance Tomography

Using voltage measurements from the boundary with a flowing electric current, ERT is a soft-field tomography method that can reconstruct the distribution of internal electrical properties of a conductive object [26,27]. In order to obtain tomographic images, ERT has to solve two sets of problems, i.e., a forward problem and an inverse problem. The forward problem solves the electrical potential u of a domain with a known distribution of conductivity σ, forming the foundation for inverse analysis. The aim of the inverse problem is to obtain the distribution of conductivity supplied to the forward operator until the difference between the computationally predicted voltage data matches the experimentally measured one with a minimum deviation. In this study, carbon ink was used to print conductive sensing skins onto concrete surfaces. Since the thickness of the sensing skins is in the order of micrometers, and the conductivity of the carbon ink is several orders of magnitude higher than that of the concrete substrate, the application of ERT to the sensing skins can be solved as two-dimensional electrical problems.

Derived from Maxwell’s equations, the forward problem is governed by a Laplacian equation as follows:

(1) ∇·(σ·∇u)=0

To apply boundary conditions to Equation (1), the complete electrode model (CEM) is employed. It is difficult to analytically solve Equation (1), so the most widely used numerical method, the finite element method (FEM), is usually adopted to solve a discrete approximation of Equation (1).

Then, the inverse problem can be formulated to minimize Equation (2) in the least-squares sense, i.e.,

(2) $$\sigma^{*}=\underset{\sigma }{\min}(\left\|V_{m}\right.{\left.-\;F(\sigma )\right\|}^{2})$$

in which $V_{m}$ is a vector of the measured boundary voltage data, and F is the forward operator that is carried out by using the FEM. For damage detection problems, the background distribution of conductivity $\sigma_{0}$ is usually known; Taylor series expansion can be used to approximate $F\left(\sigma \right)$ by centering about $\sigma_{0}$. By doing so, the nonlinear inverse problem of Equation (2) is transformed to a linear problem, which obtains the change in conductivity as follows:

(3) $${∆\sigma }^{*}=\underset{∆\sigma }{\min}\left({\left\|∆V-J∆\sigma \right\|}^{2}\right)$$

in which $∆V\;=V_{m}-F\left(\sigma_{0}\right)$, $∆\sigma =\sigma -\sigma_{0}$ and $J\;=\;\frac{\partial F\left(\sigma_{0}\right)}{\partial \sigma }$ is called the sensitivity matrix. J can be calculated by enforcing the conservation of power through the electrodes, and relating conductivity perturbations in the domain to the electrode voltage perturbations as shown in Equation (4):

(4) $${\left[J\right]}_{\mathrm{ij}}=-\int \sum_{i=1}^{2}{\left(\nabla u\right)}_{i}{\left(\nabla u^{*}\right)}_{j}\mathrm{dxdy}$$

in which $u_{i}$ and $u_{j}$ are the voltages when the ith and jth electrode pair is carrying a unit current, respectively. 

Generally speaking, reconstructing the change in conductivity by directly inversing the matrix from Equation (3) is not straightforward, because J is near singular and the least-squares inverse problem is ill-posed due to measurement noise and modeling error. Mathematically, to obtain a bounded solution, regularization approaches are usually needed for solving Equation (3). Here, the well-known Tikhonov regularization is employed to solve the ill-posed problem as follows:

(5) $$∆\sigma^{*}=\arg\underset{∆\sigma }{\min}\left({\left\|∆V-J∆\sigma \right\|}^{2}+{\left\|L∆\sigma \right\|}^{2}\right)$$

in which the second term in the right side of Equation (5) is a regularization term, and L=λI is the regularization matrix, where I is the unit matrix and λ is the regularization parameter that should be determined appropriately. The solution to Equation (5) can be obtained with the following:

(6) $$∆\sigma^{*}={\left(J^{T}J\;+\;\lambda^{2}I\right)}^{-1}J^{T}∆V$$

Nonetheless, the Tikhonov regularization is an L2 norm-based regularization method; it usually overly smooths the solution and blurs the boundary of the damage, reducing the accuracy of crack identification. Thus, in this study, total variation (TV) regularization that protects edges and details from loss is employed to solve the ERT inverse problem [28]. Its unconstrained form can be written as follows:

(7) $$∆\sigma^{*}=\arg\underset{∆\sigma }{\min}\left(\frac{\gamma }{2}{\left\|J∆\sigma -∆U\right\|}_{2}^{2}+\mathrm{TV}\left(∆\sigma \right)\right)$$

where γ is a non-negative regularization parameter, and $\mathrm{TV}\left(∆\sigma \right)=\int \Omega \left|\nabla \left(∆\sigma \right)\right|d\Omega$ is the TV of Δσ.

By introducing an auxiliary variable v=∇(Δσ), Equation (7) can be written as the following unconstrained problem:

(8) $$\left(∆\sigma^{*},v^{*}=\arg\underset{∆\sigma ,v}{\min}\frac{\lambda }{2}{\left\|J∆\sigma -∆U\right\|}_{2}^{2}+\frac{\beta }{2}{\left\|v-\nabla \left(∆\sigma \right)\right\|}_{2}^{2}+{\left\|v\right\|}_{1}\right)$$

where β is a penalty factor.

According to the Bregman formula, Equation (8) can be solved iteratively in this way:

(9) $$\left\{\begin{array}{c} \left(∆\sigma^{\left(k+1\right)},v^{\left(k+1\right)}\right)=arg\underset{∆\sigma ,v}{\min}\left|v\right|+\frac{\gamma }{2}{\left\|J∆\sigma -∆U\right\|}_{2}^{2}+\frac{\beta }{2}{\left\|v-\nabla \left(∆\sigma \right)-s^{\left(k\right)}\right\|}_{2}^{2}\\ s^{\left(k+1\right)}=s^{\left(k\right)}+\nabla \left(∆\sigma^{\left(k+1\right)}\right)-v^{\left(k+1\right)} \end{array}\right.$$

Solving the first optimization problem in Equation (9) needs to be carried out in two steps. First, fix v and obtain the solution of ∆σ by using the Gauss–Seidel method or Fourier transform as follows:

(10) $$∆\sigma^{\left(k+1\right)}\;=\arg\underset{∆\sigma }{\min}\frac{\gamma }{2}{\left\|J∆\sigma -∆U\right\|}_{2}^{2}+\frac{\beta }{2}{\left\|v^{\left(k\right)}-\nabla \left(∆\sigma \right)-s^{\left(k\right)}\right\|}_{2}^{2}$$

Then, fix ∆σ and obtain the value of v by using the Shrink operator as follows:

(11) $${\;v}^{\left(k+1\right)}\;=\arg\underset{v}{\min}{\left\|v\right\|}_{1}+\frac{\beta }{2}{\left\|v-\nabla \left(∆\sigma^{\left(k+1\right)}\right)-s^{\left(k\right)}\right\|}_{2}^{2}$$

The above method for solving the TV regularization problem using the split Bregman method is called the TV-SB algorithm [29,30]. Its steps are listed in Algorithm 1.
**Algorithm 1:** Steps of TV-SB Algorithm.Step 11.1 Set iteration number k=1 1.2 Initialization: $∆\sigma^{\left(0\right)}=∆U$, $v^{\left(0\right)}=0$, $s^{\left(0\right)}=0$Step 22.1 If the condition for termination is not met, go to Step 3 2.2 If the condition for termination is met, go to Step 5Step 33.1 Use Equation (10) to obtain $∆\sigma^{\left(k+1\right)}$
3.2 Use Equation (11) to obtain $v^{\left(k+1\right)}$
3.3 Set $s^{\left(k+1\right)}=s^{\left(k\right)}+\nabla \left(∆\sigma^{\left(k+1\right)}\right)-v^{\left(k+1\right)}$Step 44.1 Set iteration number k=k+1, go to Step 2Step 55.1 End algorithm, output $∆\sigma^{\left(k\right)}$

## 3. Experimental Study

### 3.1. Experimental Set-Up

**Figure 1:**
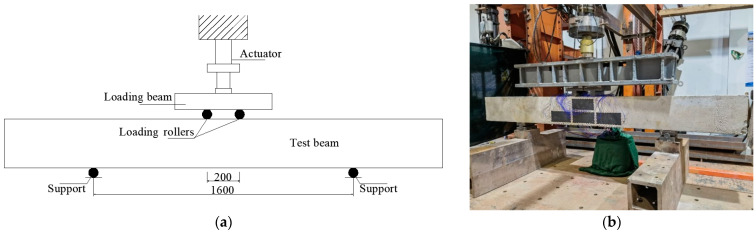
Fatigue test set-up for reinforced concrete beam: (**a**) illustration of loading scheme (Unit: mm); (**b**) scene of fatigue test.

In the experimental studies, a reinforced concrete beam with dimensions of 200 mm × 300 mm × 2700 mm was considered. As illustrated in Figure 1, the concrete beam was subjected to a 4-point bending load, and constant amplitude fatigue cycles were applied to study the initialization and evolution of cracks. Sinusoidal wave-shaped loads were applied to the beam by using the hydraulic actuator during the tests, the loading frequency was 1.0 Hz, and the maximum and minimum loads were 90 kN and 18 kN, respectively.

**Figure 2:**
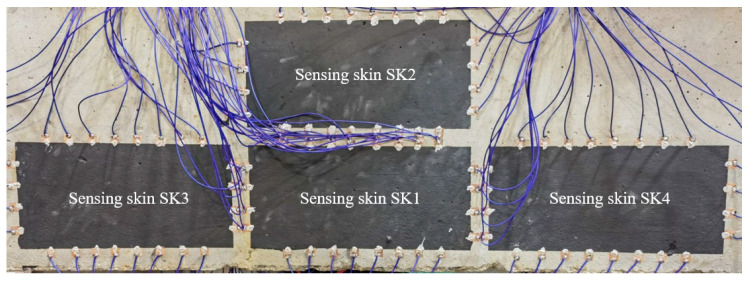
Screen-printed carbon-ink-based sensing skins on the surface of concrete.

To detect the occurrence of cracks and monitor their growth, carbon ink was coated on the surface of the concrete beam as sensing skins using a manual screen-printing process. As shown in Figure 2, four rectangular sensing skins (designated as SK1~SK4) were printed on the middle area of one lateral side of the beam, where cracks were expected to appear. For each sensing skin, the dimensions were set as 225 mm × 125 mm. LN-GCI-III conductive carbon ink produced by Lite Nano Technology Co., Ltd. (Jining, Shangdong, China). was used as a raw material, and its surface resistivity was about 10~15 Ω/mm^2^. According to the producer, a very small amount of graphene is doped to the ink to increase the conductivity. The carbon ink has a black paste appearance and was uniformly integrated on the surface of the concrete through silk printing. It was cured at ambient temperature for 8 h. After the solvent in the carbon ink evaporated, the conductive fillers came close to each other to form a conductive network. For each sensing skin, 24 copper foil electrodes were then evenly placed at designated positions on their boundaries using Type 3703 conductive silver paste produced by Xinwei New Materials Co., Ltd. (Shenzhen, Guangdong, China). The surface resistivity of the conductive silver paste was about 2.5 × 10^−3^ Ω/mm^2^, and it was cured at ambient temperature for 24 h.

**Figure 3:**
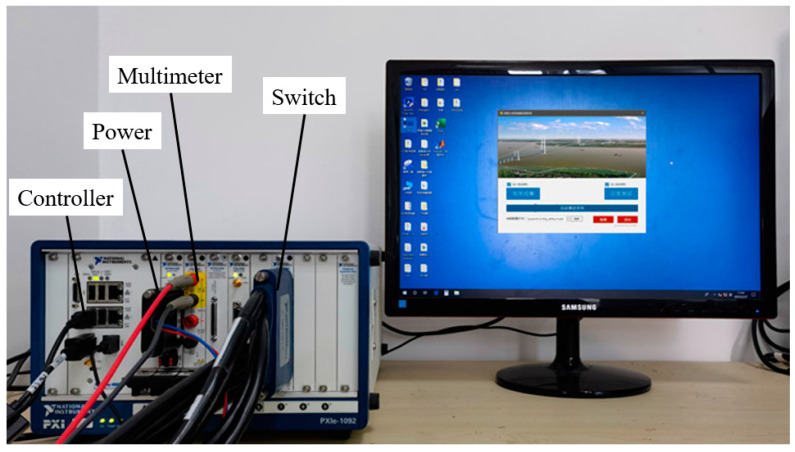
ERT testing system.

**Figure 4:**
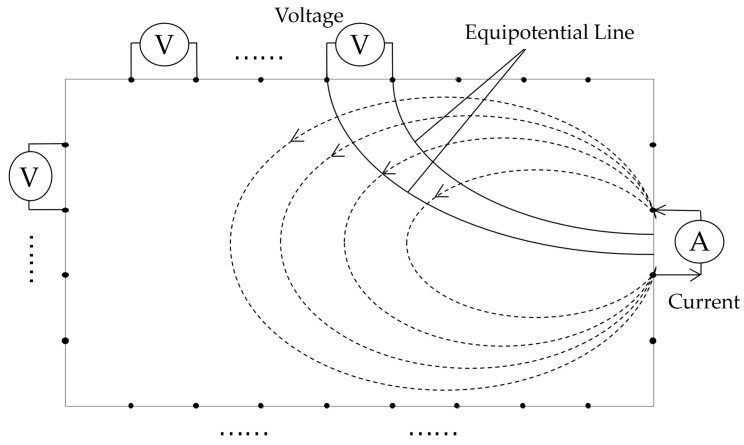
Illustration of current excitation and data acquisition.

For ERT testing, the electrodes of the sensing skins were connected to a set of data acquisition systems developed based on the PXI platform, as shown in Figure 3. It mainly consisted of a controller (NI-PXIe 8880), a precision power board (NI-PXIe 4136), a high-precision multimeter board (NI-PXIe 4081), and a channel switching board (NI-PXIe 2737). During the experimental studies, at each testing interval, adjacent electrode modes for excitation and testing were selected. When a pair of adjacent electrodes was excited by a current, the voltage value between adjacent electrodes unrelated to the excitation was recorded. This excitation and acquisition pattern is illustrated in Figure 4. The excitation current was 100 mA, 504 (24 × 21) adjacent boundary voltage values were measured for each sensing skin, and a total of 2016 data points were obtained from the four sensing skins.

### 3.2. Results for Fatigue Crack Evolution 

To accelerate the occurrence of cracks, before fatigue tests, a static load of 90 kN was applied, and the first initial crack appeared in the middle, with a maximum crack width of 0.10 mm and crack length of 61 mm. Following this initial crack, fatigue loads were applied to the concrete beam. When the fatigue test was loaded to 20,000 cycles, three new cracks appeared. In the subsequent cyclic loading process, there were a few new cracks, mainly due to the increase in the length and width of existing cracks. When loaded to 100,000 cycles, the maximum crack length reached 191 mm; when loaded to 200,000 cycles, two new cracks appeared, one of which was a diagonal shear crack with a length of 184 mm. The original cracks had evolved, and the maximum crack width reached 0.62 mm; when loaded to 290,100 cycles, two new oblique shear cracks appeared, each with a length exceeding 200 mm. In addition, two cracks intersected near the mid-span in the tensile zone of the lower part of the test beam, forming a “herringbone”-shaped crack. The maximum crack width quickly exceeded 1.50 mm, and the crack width on its symmetrical plane also exceeded 1.25 mm, basically forming a vertical crack that ran through the cross-section. At this moment, the concrete beam was considered as failed under the applied fatigue load, and the test stopped.

**Figure 5:**
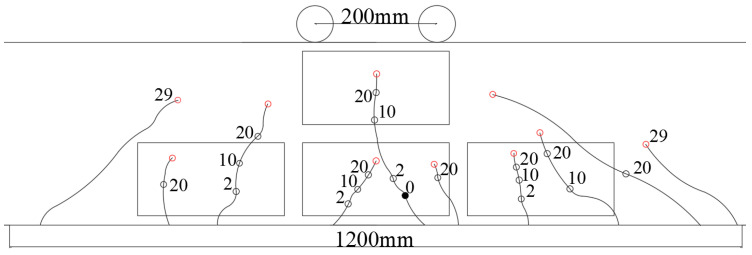
Summary of crack evolution in the concrete beam during fatigue tests (Unit: 10,000 cycles).

The evolution of cracks on the lateral surface of the concrete beam is summarized and depicted in Figure 5. The labelled numbers in the figure represent the number of fatigue cycles (unit: 10,000 cycles) when the lengths of cracks are observed. The black solid line represents the evolution trend of cracks, and the black hollow circles and marked numbers represent the location of crack evolution when the fatigue cycle loading reaches the labelled number of fatigue cycles. The black solid circle represents the initial crack occurrence and evolution location during the first static load test, the red hollow circles represent the final evolution location of the crack during fatigue failure (290,100 cycles), and the rectangular solid line indicates the monitoring range of crack damage.

### 3.3. Results for Crack Detection

**Figure 6:**
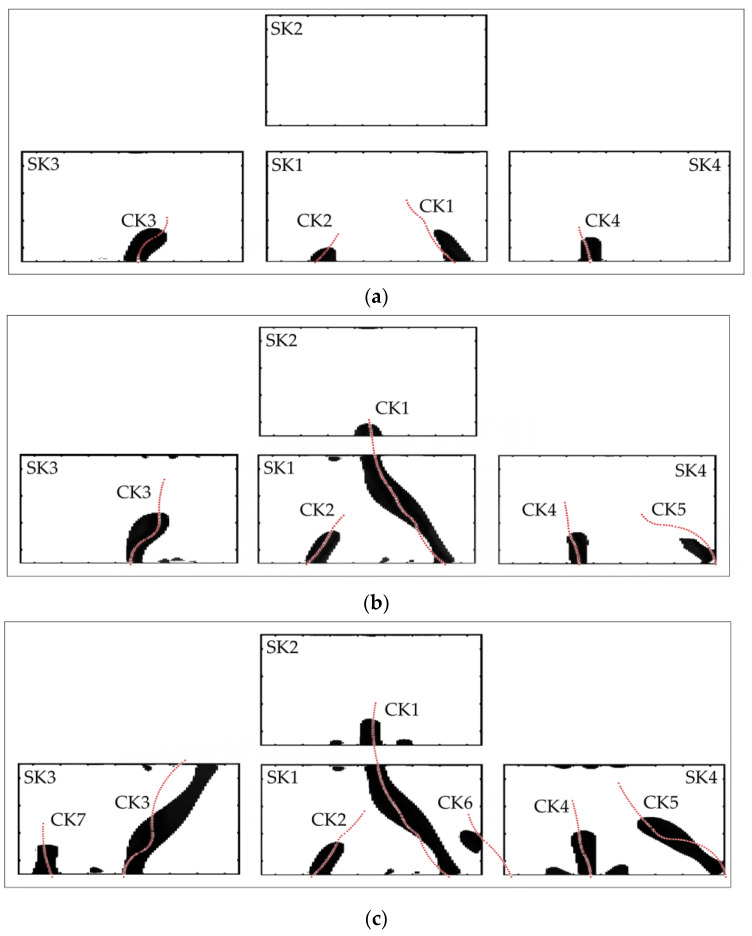
Reconstructed tomographic images for fatigue cracks after different cycles: (**a**) after 20,000 cycles; (**b**) after 100,000 cycles; (**c**) after 200,000 cycles (dashed red lines represent actual cracks observed in experiments).

Referring to the process of crack evolution in fatigue tests of the reinforced concrete beam, screen-printed sensing skins and ERT were used to image and identify the crack situation at fatigue intervals of 20,000, 100,000, and 200,000 cycles, respectively. The distribution of cracks in fatigue tests was relatively complex, with multiple cracks occurring and developing in the same monitoring area. If traditional dynamic ERT algorithms are used, that is, damage identification at different times is based on the initial “intact state” for damage image reconstruction, due to the overlapping interference of cracks, the limited number of tested boundary voltage data is insufficient to provide feedback on all the cracks. Therefore, for each sensing skin, the method of “phased identification and superposition” is adopted, which means that by subtracting different states, the cracks of each time period are identified separately and then stacked and superposed together. Specifically, before static and fatigue loads were applied, small currents were injected into the sensing skins and corresponding voltage measurements were collected according to the selected adjacent electrode mode to obtain a baseline dataset from the pristine state; then, at fatigue intervals of 20,000 cycles, the same testing procedure was performed to collect a voltage dataset, and by comparing with the baseline dataset, the TV-SB algorithm was used to reconstruct changes in conductivities for sensing skins to generate information about cracks at this interval. At the fatigue intervals of 100,000 and 200,000 cycles, the testing procedure was still the same; however, in the TV-SB algorithm, the voltage dataset was obtained at the last time period, i.e., fatigue intervals of 20,000 and 100,000 cycles were used as the “baseline” dataset, respectively. Since ERT images were only used to indicate the locations and sizes of the cracks, for convenience, the reconstructed tomographic images were first binarized into black and white images and then superimposed together. Figure 6 shows the reconstruction results of fatigue cracks under different fatigue cycles after collected boundary voltages were processed by using the TV-SB regularization algorithm; the actual cracks summarized in Figure 5 were also superimposed on the images for comparison by using dashed red lines.

As shown in Figure 6a, a total of four cracks (designated as CK1~CK4) appeared when the fatigue loading reached 20,000 cycles. The reconstructed tomographic image of the change in conductivity of the sensing skins can accurately display the location and direction of each crack that appears at this stage. In fact, the width of these new cracks is relatively small and it is difficult to detect them through visual inspection. However, it can be seen that once new cracks appear on the lateral surface of the concrete beam, they can crack the sensing skins and cause significant changes in the distribution of conductivity, allowing each new crack to be detected via the sensing skins and ERT in a timely manner.

From Figure 6b, it can be seen that when the fatigue loading reached 100,000 cycles, the original four cracks continued to grow and a new crack (designated as CK5) appeared. The initial crack CK1 within the range of sensing skin SK1 penetrated its monitoring area and evolved into sensing skin SK2. This crack was accurately identified, and reconstruction results were composed of a part in the SK1 area and another part in the SK2 area. Compared to Figure 6a, it can be seen that for the other three cracks, i.e., CK2, CK3, and CK4 in SK1, SK3, and SK4, the reconstructed tomographic image of the conductivity changes also reflected their growth, though the identified lengths showed a certain deviation from the true lengths of the cracks. There was a newly emerged crack CK5 in the right edge of SK4 at this stage; it was also clearly indicated in the tomographic image and its location and direction were accurately identified.

From Figure 6c, it can be seen that when the fatigue loading reached 200,000 cycles, the original five cracks in Figure 6b continued to grow and two new cracks (designated as CK6 and CK7) appeared. The initial crack CK1 in SK1 had already penetrated the monitoring area into SK2; its new growth in SK2 introduced new conductivity changes in SK2. The growth of cracks CK3, CK4, and CK5 in the monitoring areas of SK3 and SK4 was successfully reflected by conductivity changes in these two areas. A new crack CK6 emerged in the area between SK1 and SK4; its tip extended into the monitoring area of SK1, which was caught by the tomographic image in SK1. Another new crack CK7 emerged in the monitoring area of SK3; it was successfully identified by the tomographic image in SK3. Thus, it can be seen from the figure that with measurement data from sensing skins, the reconstructed tomographic images developed by using the TV-SB algorithm provided information about the number, location, and growth direction of existing and newly emerged cracks. However, similar to the identification results in Figure 6b, due to the dispersive nature of ERT, the lengths of cracks identified from the tomographic images were not very accurate, which needs further studies to improve.

### 3.4. Discussion

From the above experimental results, it can be seen that sensing skins fabricated by using carbon ink can detect cracks at an early stage during the fatigue process, and the growth of cracks can be monitored by using the ERT method to some extent. According to the authors’ knowledge, this is the first time that carbon ink has been applied to the health monitoring of concrete structures through screen printing. Carbon ink can be produced on an industrial scale, which greatly enriches the sensing materials required for monitoring large-scale structures such as concrete, and provides an important reference for the application of other similar low-cost materials in civil engineering.

However, there were also some shortcomings in our work. It can be observed from the results that although the dynamic ERT algorithm based on TV regularization can detect crack growth at different fatigue stages, generally, it is only qualitative. It cannot provide very accurate information about the width and length of the cracks. In particular, it is necessary to develop quantifiable indicators to quantitatively determine the error of crack identification and provide a basis for decision-making. We will strengthen our work in this area in the future.

## 4. Conclusions

In this paper, an experimental work in crack detection and imaging for concrete structures using screen-printed sensing skins and ERT is presented. Carbon ink is employed as a conductive material to be screen-printed on the surface of concrete structures to form sensing skins, and an ERT testing system is used to excite small currents in sensing skins and measure related voltage data from their boundaries. By processing voltage datasets with a TV regularization-based algorithm, changes in conductivity of the sensing skins caused by cracks and their growth are reconstructed to provide information about the cracks. 

Experimental studies on a concrete beam subjected to fatigue tests have demonstrated that carbon ink is a good sensing material for the fabrication of printed sensing skins on the surface of concrete structures, and it is feasible and effective to use a combination of printed sensing skins and ERT to detect cracks and their growth. This TV-SB algorithm can reconstruct the distribution of conductivity changes in printed sensing skins to capture the occurrence of cracks in a timely manner and provide information about the location and direction of the cracks, achieving crack identification in concrete structures. However, quantitatively identifying the widths and lengths of cracks is still challenging; the currently identified lengths of cracks from tomographic images show a certain deviation from the true lengths of the cracks. This requires further investigation for damage identification in concrete structures using screen-printed sensing skins and ERT.

## Data Availability

Not applicable.
